# Exploring the Multi-Faceted Effects of Berberine in Ameliorating Diastolic Dysfunction in Rats with Heart Failure with Preserved Ejection Fraction

**DOI:** 10.3390/ijms26104847

**Published:** 2025-05-19

**Authors:** Yu Mu, Jing Geng, Chilu Liu, Shuang Jiang, Yanxing Han, Jiandong Jiang, Yuhong Wang

**Affiliations:** 1 State Key Laboratory of Bioactive Substances and Function of Natural Medicine, Institute of Materia Medica, Chinese Academy of Medical Sciences and Peking Union Medical College, Beijing 100050, China; 2 Institute of Medicinal Biotechnology, Chinese Academy of Medical Sciences and Peking Union Medical College, Beijing 100050, China

**Keywords:** berberine, heart failure, diastolic dysfunction, inflammation, oxidative stress, cardiovascular fibrosis, endothelial dysfunction

## Abstract

Heart failure with preserved ejection fraction (HFpEF), marked by cardiac diastolic dysfunction, contributes to half of all heart failure cases globally and poses a significant public health challenge. Effective therapies for HFpEF are rare, largely due to its complex and heterogeneous pathophysiology, which often involves multiple comorbidities. Berberine (BBR), an isoquinoline alkaloid, has demonstrated beneficial effects on multiple metabolic and cardiovascular disorders; however, its impact on cardiac diastolic dysfunction in HFpEF remains poorly understood. In this study, we utilized a rat model of HFpEF induced by a sustained high-fat/high-sucrose (HFHS) diet to explore the impact and mechanisms of BBR on diastolic dysfunction. The results revealed that BBR administration effectively alleviated cardiac diastolic dysfunction and alleviated extracardiac comorbidities, including increased weight, impaired glucose tolerance, hypercholesterolemia and hypertension, in rats fed an HFHS diet. Furthermore, BBR mitigated myocardial inflammation, oxidative stress, microvascular endothelial dysfunction, and notably restored the disturbed NO-cGMP-PKG pathway. Additionally, BBR reduced myocardial fibrosis and inhibited the abnormally activated TGF-β/Smads signaling. Moreover, BBR attenuated the systemic inflammation and corrected immune dysregulation in an HFHS diet-fed rats. Our study suggests that BBR exhibits multi-beneficial effects in the prevention and management of HFpEF, demonstrating its potential as a holistic therapeutic candidate for HFpEF.

## 1. Introduction

Heart failure (HF) poses a substantial clinical and public health challenge, with nearly half of the cases attributed to HF with preserved ejection fraction (HFpEF), which is marked by cardiac diastolic dysfunction with normal ejection fraction (EF) values [1]. Despite its high prevalence and poor prognosis with five-year mortality rate exceeding 50%, conventional pharmacological therapies for HF with reduced ejection fraction (HFrEF) have demonstrated limited efficacy in improving clinical outcomes for patients with HFpEF [2,3]. This highlights an urgent medical need for effective treatments to HFpEF.

Cardiac diastolic dysfunction constitutes a core pathological feature in the development of HFpEF, with low-grade inflammation being a key mechanism leading to this dysfunction. Patients with HFpEF often present with numerous extracardiac inflammatory comorbidities, notably obesity, hypertension, type 2 diabetes and aging [4]. In recent years, novel therapeutic agents, including glucagon-like peptide-1 (GLP-1) receptor agonists and sodium-glucose cotransporter 2 (SGLT2) inhibitors, have shown promising results in improving symptoms and prognosis in patients with HFpEF [5,6]. Rising evidence indicates that, beside hypoglycemic effect, SLTG2 inhibitors and GLP-1 receptor agonists have a systemic impact via indirectly targeting heart by regulating inflammation, oxidative stress, nitric oxide (NO) bioavailability, cardiac fibrosis, and other mechanisms [7,8,9]. Furthermore, the anti-inflammatory properties of SGLT2 inhibitors and GLP-1 receptor agonists may account for their cardiovascular benefits that extend beyond glucose-lowering effects [9,10]. However, their application may be constrained by a limited target population, high drug costs, and potential adverse effects, such as urinary tract infections. In spite of this, these findings suggest a novel and intriguing therapeutic strategy for HFpEF.

Plant-based medicines have a long history in the treatment of chronic HF. Berberine (BBR), a bioactive isoquinoline alkaloid derived from the medicinal plant *Coptis chinensis*, is well known for its antibacterial and antidiarrheal properties [11]. It has also demonstrated significant benefits for cardiovascular disease due to its pleiotropic effects, including the regulation of glucose homeostasis and lipid profile, attenuation of inflammatory responses and oxidative damage, and inhibition of fibrotic processes [12,13]. Clinical investigations have established the safety and clinical effectiveness of BBR in the treatment of HF patients [14]. Additionally, BBR holds promise as a therapeutic agent for systemic inflammation, as it has shown therapeutic potential in both prophylactic and therapeutic interventions for chronic inflammation associated disease, including atherosclerosis and/or non-alcoholic fatty liver disease by controlling inflammation [15]. These findings underscore its promise as a novel therapeutic candidate for HFpEF. Although a recent study has shown that BBR mitigated diastolic dysfunction in a murine HFpEF model possibly by inhibiting mitochondrial fragmentation [16], the mechanism by which BBR improves cardiac diastolic dysfunction still needs to be further explored.

Previous investigations have established that chronic exposure to calorie-dense diets can impair cardiac diastolic function and contribute to the progression of HFpEF [17,18]. This study aims to comprehensively assess the impact of BBR on diastolic dysfunction in rats with HFpEF caused by a sustained high-fat, high-sucrose (HFHS) diet, hoping to establish a theoretical foundation for its potential translation into clinical practice in the future.

## 2. Results

### 2.1. BBR Ameliorates Cardiac Diastolic Dysfunction, Hypertrophy, and Extracardiac Comorbidities in HFHS Diet-Fed Rats

**Figure 1:**
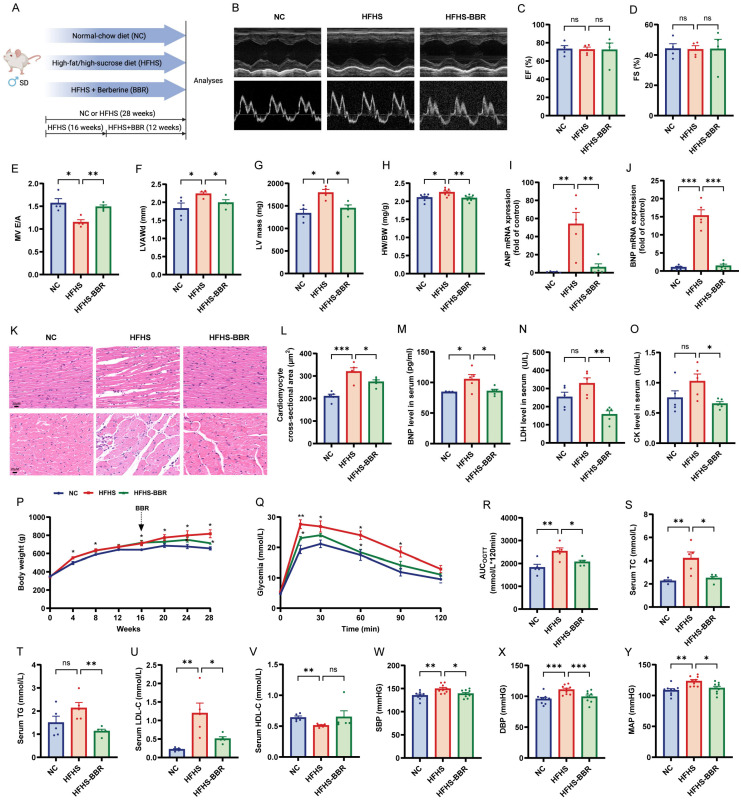
BBR ameliorates cardiac diastolic dysfunction, hypertrophy and metabolic disorders in HFHS diet rats. (**A**) Schematic diagram of experiments in rats. (**B**) Representative M-mode and pulse-wave Doppler echocardiograms. (**C**–**G**) Statistical analyses of left ventricular ejection fraction (EF), left ventricular fractional shortening (FS), mitral inflow E and A wave ratio (MV E/A), LV anterior wall thickness (LVAW) and LV mass. (**H**) Statistical analysis of the heart weight (HW) to body weight (BW) ratio. (**I**) ANP and (**J**) BNP mRNA expression levels in myocardium. (**K**) HE staining and (**L**) quantitative analysis of cardiomyocyte cross-sectional area (CSA). Scale bars: 20 μm. (**M**–**O**) Serum BNP, lactate dehydrogenase (LDH), and creatine kinase (CK) levels. (**P**) Body weight. (**Q**) Oral glucose tolerance test (OGTT) and (**R**) OGTT area under the curve (AUC). (**S**–**V**) Serum levels of total cholesterol (TC), triglyceride (TG), low-density lipoprotein cholesterol (LDL-C) and high-density lipoprotein cholesterol (HDL-C). (**W**–**Y**) Systolic blood pressure (SBP), diastolic blood pressure (DBP), and mean arterial pressure (MAP). Data are expressed as the mean ± SEM. * *p* < 0.05, ** *p* < 0.01, *** *p* < 0.001; ns, not significant.

Echocardiography results showed that rats fed an HFHS diet had similar ejection fraction (EF) and fractional shortening (FS) values to normal control (NC) rats. However, they exhibited evident cardiac diastolic dysfunction, evidenced by a decreased mitral E/A ratio (Figure 1B–E), and cardiac hypertrophy, indicated by increases in left ventricular anterior wall thickness (LVAW), LV mass, and heart weight to body weight ratio (HW/BW) (Figure 1F–H). Furthermore, myocardial mRNA expressions levels of ANP and BNP were significantly increased in HFHS diet-fed rats (Figure 1I,J). Histological analysis using H&E staining showed disordered cardiomyocytes, infiltration of inflammatory cells, and increased cardiomyocyte cross-sectional area (CSA) (Figure 1K,L). Additionally, serum levels of BNP, lactate dehydrogenase (LDH) and creatine kinase (CK) were elevated (Figure 1M–O). Importantly, BBR administration significantly mitigated these cardiac abnormalities, indicating a protective effect against cardiac injury and improvement in diastolic function. Moreover, BBR reduced body weight, improved glucose tolerance, regulated blood lipid levels, and decreased blood pressure in HFHS diet rats (Figure 1P–Y), suggesting the mitigation of associated extracardiac comorbidities.

### 2.2. Network Pharmacology Predicates the Potential Mechanisms of BBR in the Treatment of HFpEF

**Figure 2:**
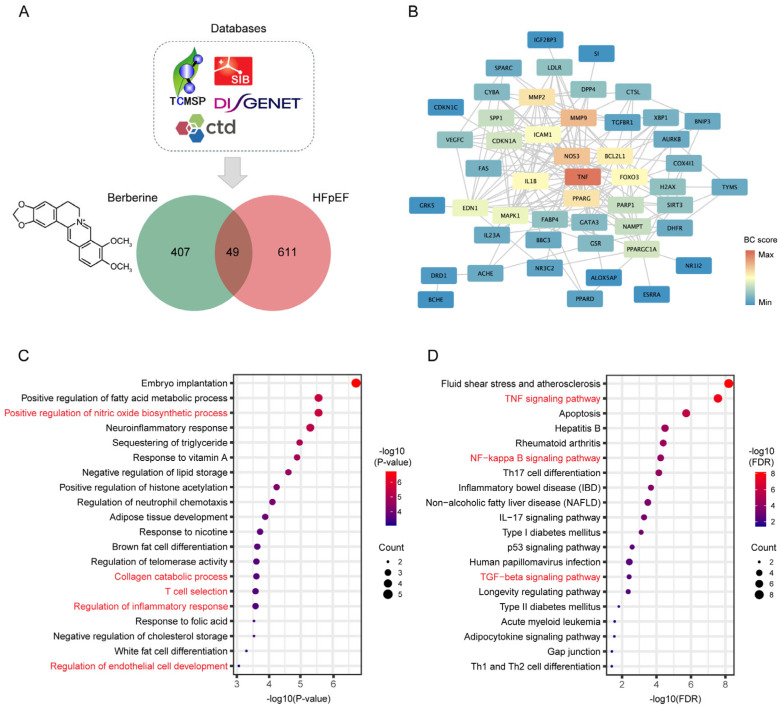
Network pharmacology predicates the potential mechanisms of BBR in the treatment of HFpEF. (**A**) Venn diagram of 49 common target genes of BBR and HFpEF. (**B**) The protein–protein interaction (PPI) network of 49 common targets of BBR and HFpEF. (**C**) GO enrichment bubble plots of BBR and HFpEF related pathways (**D**) KEGG enrichment bubble plots of BBR and HFpEF related pathways.

Given BBR’s observed therapeutic benefits in diastolic dysfunction, we conducted network pharmacology to investigate the potential mechanisms of BBR in HFpEF treatment. From various databases, we identified 49 common targets for BBR and HFpEF (Figure 2A). To obtain key regulators among these genes, the protein–protein interaction (PPI) network was established using the STRING database and graphically represented using Cytoscape visualization software (https://cytoscape.org). Based on the betweenness centrality scores of PPI network (Figure 2B and Table S3), we selected 20 key genes: TNF, PPARGC1A, MMP9, NOS3, ACHE, CDKN1A, BCL2L1, FOXO3, PPARG, EDN1, IL1B, MMP2, DRD1, DPP4, NAMPT, PARP1, FABP4, MAPK1, ICAM1, and H2AX. Subsequently, we performed GO and KEGG analysis on these targets, which suggest that the therapeutic effects of BBR in HFpEF may be closely linked to the positive regulation of nitric oxide biosynthetic process, collagen catabolic process, T cell selection, regulation of inflammatory response, and regulation of endothelial cell development (Figure 2C, Table S4). Furthermore, our finding implicated the TNF, NF-κB and TGF-β pathways as underlying signaling mechanisms (Figure 2D, Table S5). Based on these results and literature reviews [1,18,19,20], we identified several pathological processes associated with HFpEF that may be modulated by BBR, including inflammation, oxidative stress, myocardial interstitial fibrosis, microvascular endothelial dysfunction and immune dysregulation. Subsequently, we conducted further investigations to elucidate the potential mechanisms through which BBR may exert its therapeutic effects against HFpEF.

### 2.3. BBR Alleviates Myocardial Inflammation and Microvascular Inflammation

**Figure 3:**
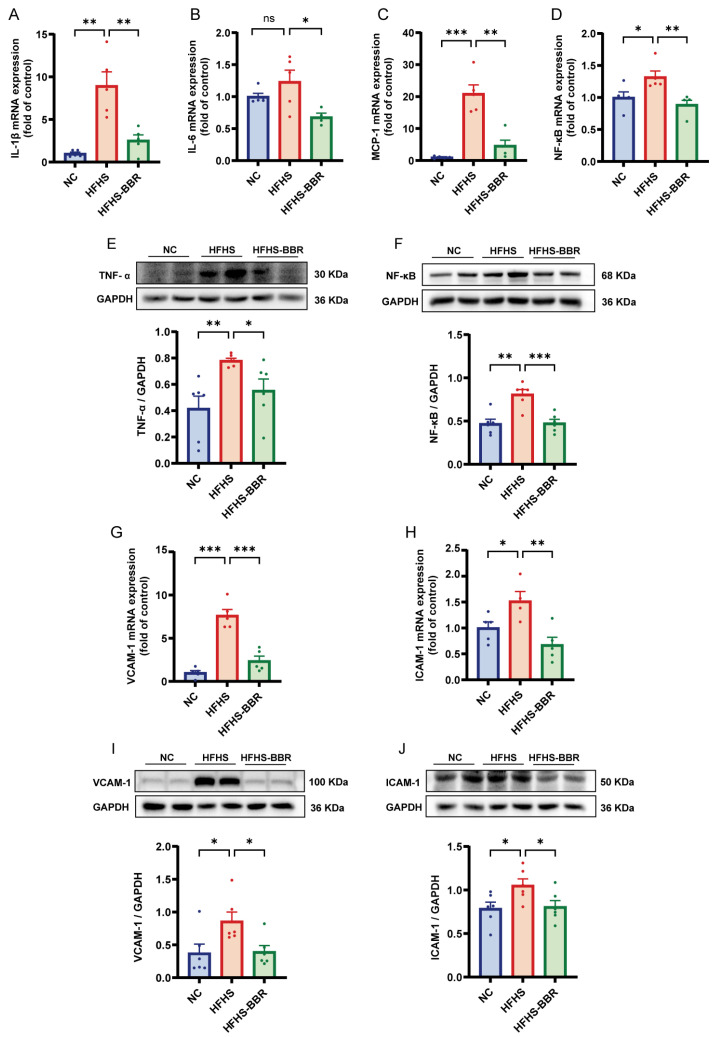
BBR alleviates myocardial inflammation and microvascular inflammation in HFHS diet rats. (**A**–**D**) Statistical analyses of IL-1β, IL-6, MCP-1 and NF-κB mRNA expression levels in myocardium. (**E**,**F**) Representative immunoblots and quantitative analysis of TNF-α and NF-κB protein expression levels. (**G**,**H**) Statistical analyses of VCAM-1 and ICAM-1 mRNA expression levels in myocardium. (**I**,**J**) Representative immunoblots and quantitative analysis of VCAM-1 and ICAM-1 protein expression levels. Data are expressed as the mean ± SEM. * *p* < 0.05, ** *p* < 0.01, *** *p* < 0.001; ns, not significant.

Compared to NC rats, HFHS diet-fed rats exhibited elevated myocardium inflammation, as evidenced by increased levels of IL-1β, IL-6, MCP-1 and TNF-α (Figure 3A–C,E). NF-κB, a key regulator of gene involved in inflammation, cellular transformation, and various chronic diseases, was found to be upregulated in HFHS diet-fed rats (Figure 3D,F). Microvascular inflammation was assessed by analyzing the expression of VCAM-1 and ICAM-1, both of which were increased in HFHS diet-fed rats compared to NC rats (Figure 3G–J). Notably, BBR application significantly reduced this increase in inflammatory marker in rats fed an HFHS diet. Overall, these findings suggest that BBR inhibits both myocardial inflammation and microvascular inflammation.

### 2.4. BBR Suppresses Myocardial Oxidative Stress

**Figure 4:**
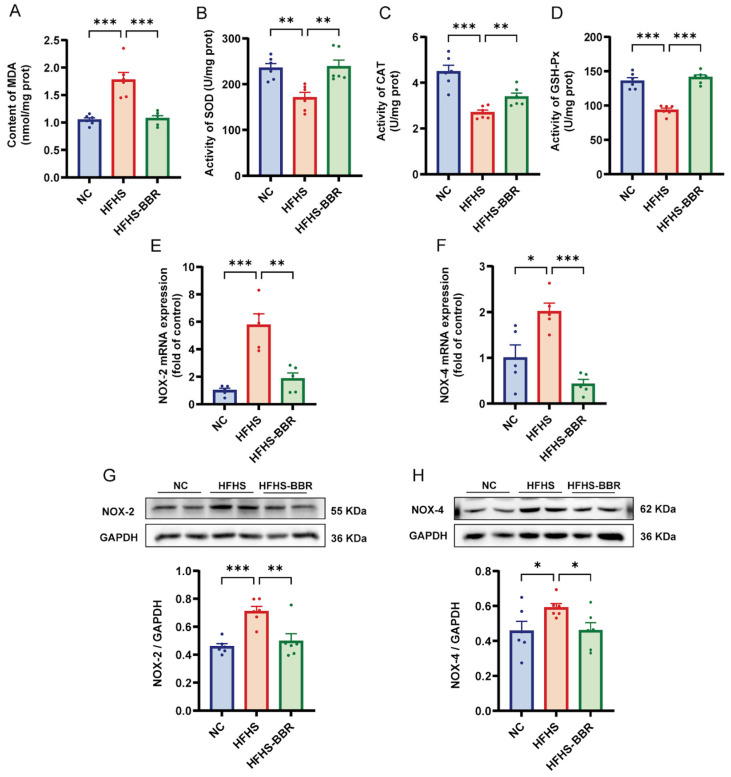
BBR suppresses myocardial oxidative stress in HFHS diet rats. (**A**) Quantitative analysis of the content of malondialdehyde (MDA) in myocardium. (**B**–**D**) Quantitative analysis of the activities of superoxide dismutase (SOD), catalase (CAT) and glutathione peroxidase (GSH-Px) in myocardium. (**E**,**F**) Statistical analyses of NOX-2 and NOX-4 mRNA expression levels in myocardium. (**G**,**H**) Representative immunoblots and quantitative analysis of NOX-2 and NOX-4 protein expression levels. Data are expressed as the mean ± SEM. * *p* < 0.05, ** *p* < 0.01, *** *p* < 0.001.

To assess myocardial oxidative stress, the levels of malondialdehyde (MDA) content and the activities of superoxide dismutase (SOD), catalase (CAT) and glutathione peroxidase (GSH-Px) were tested (Figure 4A–D). Rats fed an HFHS diet exhibited significantly elevated MDA levels along with markedly reduced activities of SOD, CAT, and GSH-Px when compared with NC rats. Notably, BBR treatment effectively counteracted these HFHS-induced oxidative disturbances. Furthermore, the mRNA and protein expression levels of NOX-2 and NOX-4 were upregulated in HFHS diet-fed rats (Figure 4E–H). Importantly, BBR administration significantly reversed these changes. Collectively, these results suggest that BBR inhibits myocardial oxidative stress and enhances myocardial antioxidant capacity in HFHS diet-fed rats.

### 2.5. BBR Diminishes Myocardial Fibrosis and Inhibits TGF-β/Smads Pathway

**Figure 5:**
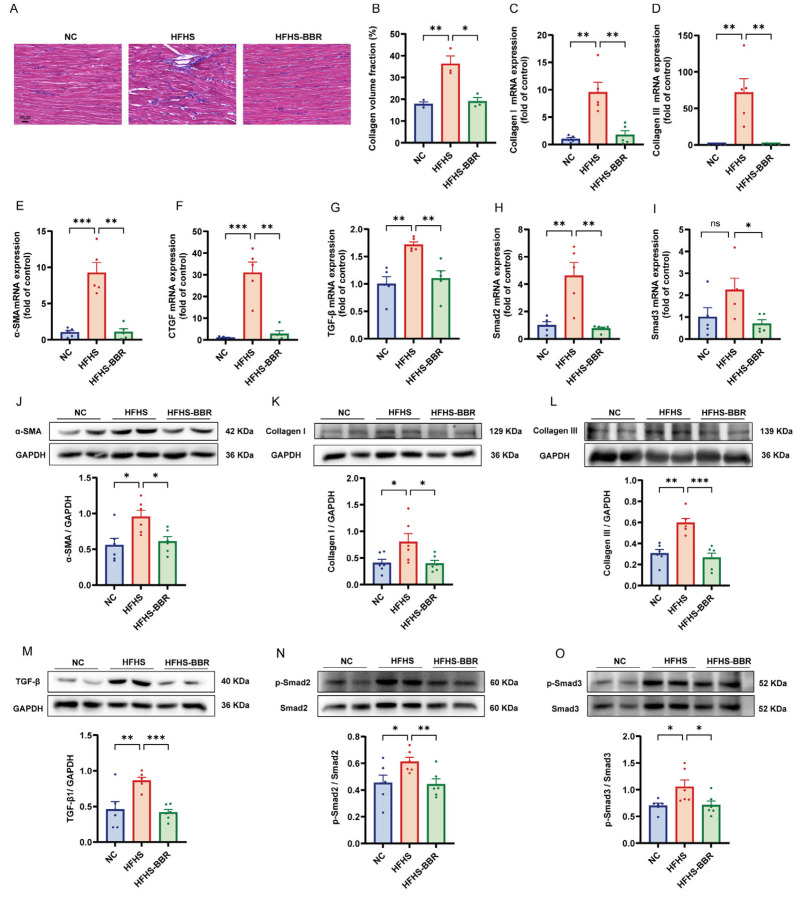
BBR diminishes myocardial interstitial fibrosis and inhibited TGF-β/Smads pathway in HFHS diet rats. (**A**,**B**) Masson staining and quantitative analysis of collagen volume fraction; Scale bar: 20 μm. (**C**–**I**) Statistical analyses of Collagen I, Collagen III, α-SMA, CTGF, TGF-β, Smad2 and Smad3 mRNA expression levels in myocardium. (**J**–**O**) Representative immunoblots and quantitative analysis of α-SMA, Collagen I, Collagen III, TGF-β, p-Smad2/Smad2 and p-Smad3/Smad3 protein expression. Data are expressed as the mean ± SEM. * *p* < 0.05, ** *p* < 0.01, *** *p* < 0.001; ns, not significant.

Masson’s trichrome staining revealed an increased collagen volume fraction in the myocardium of HFHS diet-fed rats compared to NC rats (Figure 5A,B). Additionally, the expression of cardiac fibrosis markers, namely Collagen I, Collagen III, α-SMA and CTGF, was elevated in these rats (Figure 5C–F,J–L). Notably, BBR administration significantly reduced these changes, indicating its anti-fibrotic effect. Furthermore, mRNA expression of TGF-β and Smad2/3 was upregulated in the HFHS diet rats to NC rats (Figure 5G–I). Similarly, protein expression levels of TGF-β and phosphorylation levels of Smad2 and Smad3 were elevated in these rats (Figure 5M–O). Importantly, BBR significantly decreased these alterations, suggesting that BBR attenuates myocardial fibrosis by inhibiting the TGF-β/Smads pathway.

### 2.6. BBR Restores the Impaired NO/cGMP/PKG Pathway and Reduces Cardiomyocytes Stiffness

**Figure 6:**
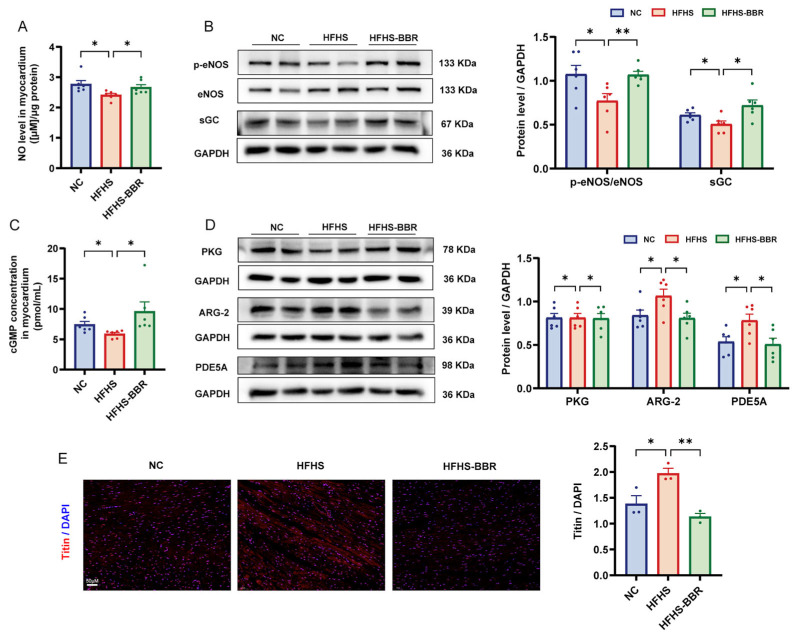
BBR activates NO/cGMP/PKG pathway in HFHS diet rats. (**A**) Quantitative analysis of total nitric oxide (NO) production in myocardium. (**B**) Representative immunoblots and quantitative analysis of p-eNOS/eNOS and sGC protein expression levels. (**C**) Quantitative analysis of cGMP concentration in myocardium. (**D**) Representative immunoblots and quantitative analysis of PKG, ARG-2 and PDE5A protein expression levels. (**E**) Immunofluorescence staining and quantitative analysis of titin (red) and DAPI (blue), Scale bar: 50 μm. Data are expressed as mean ± SEM. * *p* < 0.05, ** *p* < 0.01.

Elevated oxidative stress levels resulted in decreased nitric oxide (NO) bioavailability, which subsequently reduced soluble guanylate cyclase (sGC) activity and led to a decrease in cGMP production, the key regulator of protein kinase G (PKG) activity. To assess NO bioavailability, myocardial nitrite and nitrate concentrations were measured. HFHS diet-fed rats diet exhibited significantly lower NO production compared to NC rats (Figure 6A). Furthermore, these rats showed reduced levels of phosphorylated endothelial nitric oxide synthase (eNOS), the catalytic enzyme mediating NO biosynthesis (Figure 6B). These effects were restored upon BBR administration. Additionally, HFHS diet-fed rats had decreased expression of sGC and reduce cGMP content compared to NC rats, and BBR reversed these changes (Figure 6B–C). Moreover, PKG expression were lower in the myocardium of HFHS diet-fed rats compared to NC rats (Figure 6D), and BBR restored the PKG expression. Increase arginase (ARG) activity is known to decrease in NO production, while activation of phosphodiesterases (PDEs) promotes cGMP degradation [21]. HFHS-fed rats exhibited increased expression of ARG and PDE5A compared to NC rats, and BBR significantly decreased these elevated protein levels (Figure 6D). Overall, BBR restored the impaired NO/cGMP/PKG pathway. PKG reduces cardiomyocyte stiffness by phosphorylating titin, an essential sarcomere structural protein that regulates passive tension in cardiac myocytes [22]. Immunofluorescence analysis of the myocardium revealed the increased titin expression in HFHS diet-fed rats compared to NC rats, which decreased following BBR administration (Figure 6E). The lower titin expression indirectly reflected an increase in titin phosphorylation, resulting in reduced cardiomyocyte stiffness [23]. Thereby, BBR may reduce the cardiomyocytes stiffness by restoring the impaired NO/cGMP/PKG pathway.

### 2.7. BBR Mitigates Systemic Inflammation in HFHS Diet-Fed Rats

**Figure 7:**
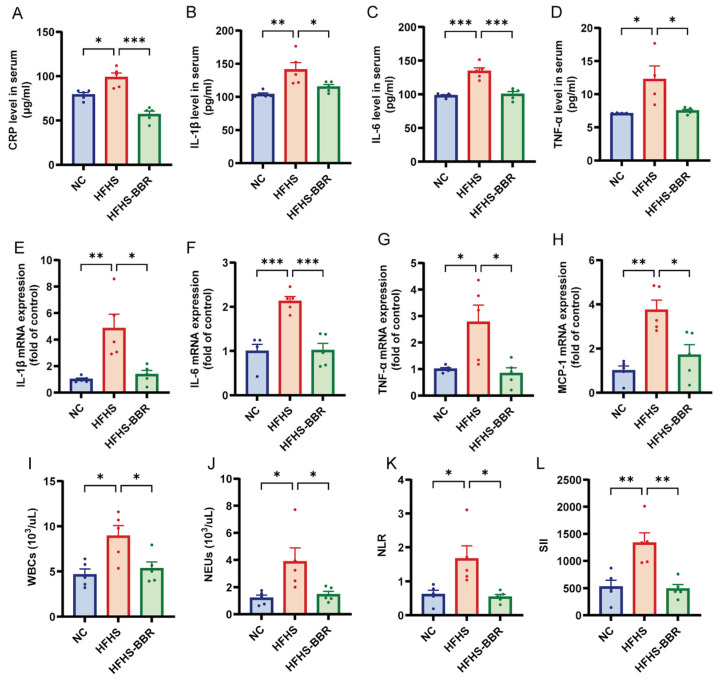
BBR ameliorates systemic inflammation in HFHS diet rats. (**A**–**D**) Quantitative analysis of serum CRP, IL-1β, IL-6 and TNF-α levels. (**E**–**H**) Statistical analyses of IL-1β, IL-6, TNF-α and MCP-1 mRNA expression levels in adipose tissue. (**I**–**L**) Statistical analyses of white blood cells (WBCs) counts, neutrophils (NEUs) counts, neutrophil to lymphocyte ratio (NLR) and systemic immune inflammation index (SII) in peripheral blood. Data are expressed as the mean ± SEM. * *p* < 0.05, ** *p* < 0.01, *** *p* < 0.001.

Calorie-dense diets trigger systemic low-grade chronic inflammation through upregulation of pro-inflammatory cytokine expression and secretion [24]. In the current study, serum levels of CRP, IL-1β, IL-6 and TNF-α were significantly elevated in HFHS diet-fed rats compared to NC rats (Figure 7A–D). Similarly, the mRNA expression of pro-inflammatory cytokines, including IL-1β, IL-6, TNF-α and MCP-1, was increased in the visceral adipose tissue of HFHS diet-fed rats (Figure 7E–H). Furthermore, hemogram analysis revealed higher levels of WBCs and NEUs, as well as elevated systemic inflammatory indices such as neutrophil to lymphocyte ratio (NLR) and systemic immune inflammation index (SII), in HFHS diet-fed rats compared to NC rats (Figure 7I–L, Table S6). Collectively, these results indicate a systemic pro-inflammatory state in rats fed an HFHS diet. Importantly, BBR administration significantly reduced the levels of inflammatory cytokines in both serum and adipose tissue, as well as the systemic inflammatory indices, indicating that BBR effectively mitigates the systemic inflammation in HFHS diet-fed rats.

### 2.8. BBR Correctes Immune Dysregulation in HFHS Diet-Fed Rats

**Figure 8:**
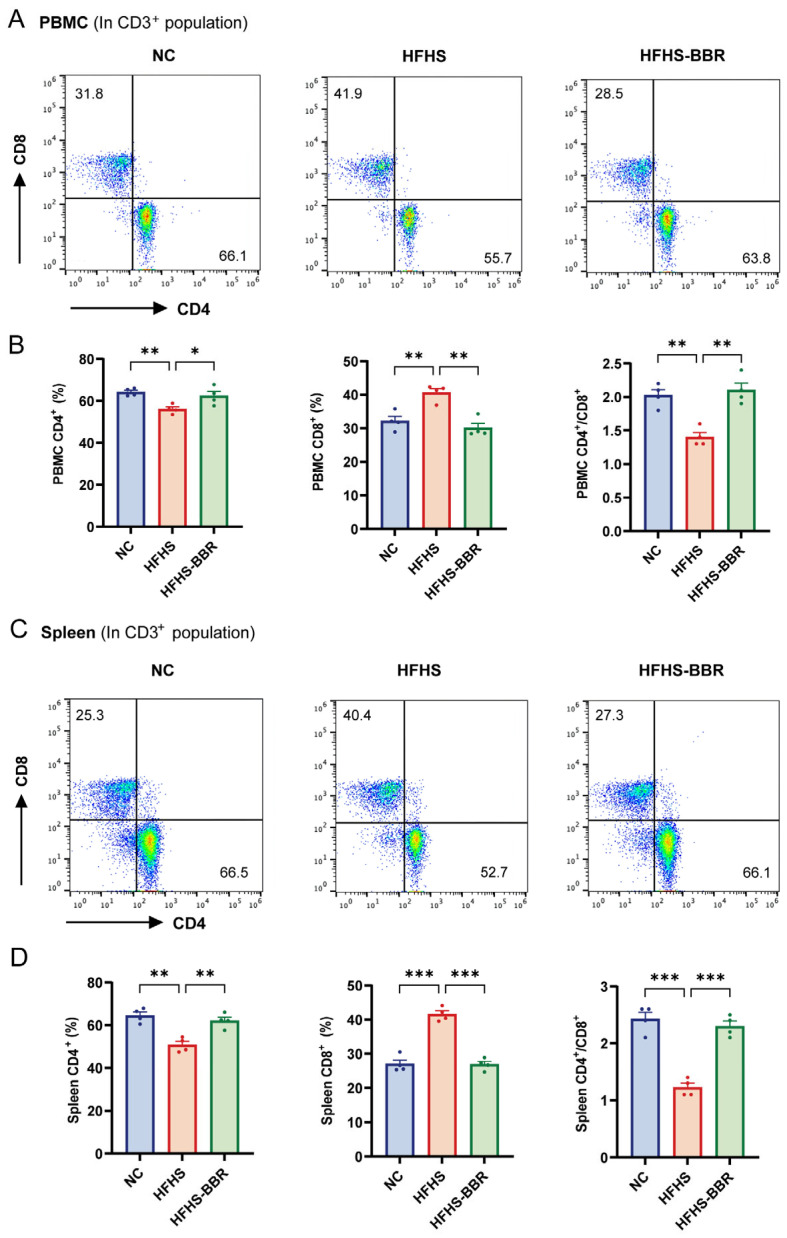
BBR regulates lymphocyte subsets disorder in HFHS diet rats. (**A**) The flow cytometric plots of CD4^+^ and CD8^+^ T cell subsets in PBMC. (**B**) Statistical analysis of the proportion of CD4^+^ T cell and CD8^+^ T cell and the CD4^+^/CD8^+^ ratio in PBMC. (**C**) The flow cytometric plots of CD4^+^ and CD8^+^ T cell subsets in spleen. (**D**) Statistical analysis of the proportion of CD4^+^ T cell and CD8^+^ T cell and the CD4^+^/CD8^+^ ratio in spleen. Data are expressed as mean ± SEM. * *p* < 0.05, ** *p* < 0.01, *** *p* < 0.001.

Calorie-dense diets have been shown to induce dysregulation of immune cells [25]. T-lymphocyte subsets proportion, particularly the CD4^+^/CD8^+^, which is traditionally used as an immune-stimulatory marker, are essential for evaluating the immune function. We then compared the proportion of CD4^+^ and CD8^+^ T cells in the CD3^+^ population of PBMC and spleen in different groups. In both PBMC and spleen, HFHS diet-fed rats exhibited a decrease in CD4^+^ T cells and an increase in CD8^+^ T cells compared to NC rats (Figure 8A–D). Furthermore, the CD4^+^/CD8^+^ ratio was significantly reduced in these tissues of HFHS diet-fed rats. Importantly, BBR administration altered the composition of T-lymphocyte subsets and significantly restored the CD4^+^/CD8^+^ ratio to a near normal levels in rats fed an HFHS diet, suggesting a potential immunoregulatory effect of BBR.

## 3. Discussion

HFpEF is a complex, multi-factorial syndrome with clinical heterogeneity and multiple comorbidities contributing to its overall presentation. The prevailing HFpEF paradigm, first proposed by Paulus W.J. and Tschöpe C in 2013 [26], posits that systemic pathological factors trigger sustained inflammatory responses and impair endothelial homeostasis, leading to microvascular impairment and subsequent disease advancement, ultimately resulting in cardiac diastolic dysfunction. This study has demonstrated that BBR exerts multi-beneficial effects on rats with HFpEF, which are relevant to the pathophysiology of HFpEF.

Cardiac inflammation and oxidative stress are known contributors to diastolic dysfunction [18,27]. BBR has demonstrated significant efficacy in attenuating inflammation and oxidative stress in various pathological conditions. Notably, BBR alleviates diabetic cardiomyopathy by inhibiting inflammasome activation [28,29], protects against doxorubicin-induced cardiomyopathy by diminishing cardiac oxidative stress [30], and alleviates myocardial ischemia-reperfusion injury by suppressing the inflammatory response and oxidative stress [31]. Our study exhibited a significant decrease in inflammation and oxidative stress in rats fed an HFHS diet following BBR treatment. The linkage between ROS and myocardial inflammation may involves the stimulation of NF-κB signaling pathways, triggering transcriptional upregulation of pro-inflammatory mediators, leading to the generation of ROS [27,32,33]. Inflammation also induces ROS production primarily through NADPH oxidase enzymes [34], resulting in elevated oxidative stress. Our results further demonstrated that BBR suppressed NF-κB signaling pathway and significantly downregulated NADPH oxidase isozyme expression, particularly NOX-2 and NOX-4, in rats fed an HFHS diet. Collectively, our findings indicate that BBR improves cardiac diastolic dysfunction by suppressing cardiac inflammation and oxidative stress.

In HFpEF, cardiac remodeling is initially driven by chronic microvascular inflammation, as supported by compelling preclinical and clinical evidence [35]. Excessive ROS generation upregulates the expression levels of vascular adhesion molecules, particularly VCAM-1 and ICAM-1 [36], facilitating myocardial infiltration by activated leukocytes, transformation of fibroblast into myofibroblasts, and the progression of myocardial fibrosis. The TGF-β/Smads signaling cascade represents one of the most crucial regulatory pathways for cardiac fibrosis formation [37]. Our results showed that BBR application inhibited microvascular inflammation, inhibited the TGF-β/Smads signaling pathway, and alleviated myocardial fibrosis in HFHS diet-fed rats. Myocardial fibrosis is a crucial contributor of cardiac diastolic dysfunction. Compelling evidence has demonstrated that reducing myocardial fibrosis would be beneficial for HFpEF patients [38]. Therefore, BBR presents profound potential for alleviating myocardial fibrosis, thereby improving diastolic dysfunction in HFpEF.

Furthermore, microvascular inflammation triggers oxidative stress and diminishes NO bioavailability, resulting in microvascular endothelial dysfunction, a pivotal event in the progression of HFpEF [36]. Additionally, sGC enzymatic activity, intracellular cGMP concentration, and PKG activation status in adjacent cardiomyocytes leads to titin hypophosphorylation, ultimately contributing to increased passive stiffness and diastolic dysfunction in HFpEF [39]. Substantial evidence has demonstrated the disruption of NO/cGMP/PKG pathway in the occurrence and development of HFpEF [40]. Importantly, our findings reveal that BBR application effectively mitigates microvascular endothelial dysfunction, rescues the disturbed NO-cGMP-PKG pathway in rats fed an HFHS diet. A previous study indicated that decreased titin expression served as an indirect indicator of elevated titin phosphorylation, leading to a decrease in cardiomyocyte rigidity [23]. BBR also effectively decreases cardiac titin expression. Thereby, BBR may reduce cardiomyocyte stiffness by restoring the disturbed NO-cGMP-PKG pathway, contributing to improve diastolic dysfunction in HFpEF.

Sustained low-grade systemic inflammation represents a key driver in the initiation and advancement of HFpEF pathophysiology [26]. Elevated levels of inflammatory biomarkers, including CRP, IL-6, IL-1β and TNF-α, have been strongly associated with disease progression and unfavorable outcomes in HFpEF [41]. A dose–response meta-analysis of randomized controlled trials has demonstrated that BBR interventions significantly reduce IL-6, TNF-α, and CRP levels in adults, indicating improved systemic inflammation status [42]. In our study, BBR administration significantly improved extracardiac pro-inflammatory comorbidities in rats with HFpEF. Furthermore, BBR significantly decreased the level of serum CRP, IL-1, IL-6, and TNF-α. Chronic inflammation is associated with adipose tissue inflammation [43]. BBR also reduced the mRNA expression of TNF-α, IL-6, IL-1β and MCP-1 in visceral adipose tissue. In addition to traditional inflammatory markers, the NLR and SII derived from peripheral blood serve as novel indices of systemic inflammation for assessing the prognosis of patients with HFpEF [41,44]. Our study also revealed that BBR significantly lowered NLR and SII in HFHS diet-fed rats. These findings show that BBR suppresses systemic low-grade inflammation in rats with HFpEF.

Multiple lines of evidence suggest that immune dysregulation is linked to the pathology and progression of HFpEF. Alteration of T-lymphocyte subsets, particularly the CD4^+^/CD8^+^ ratio, has shown predictive potential for HF in clinical and experimental studies [45]. A recent study by Cao et al. reported a decreased peripheral CD4^+^/CD8^+^ in elderly patients with chronic HF, and immunomodulatory agents administration significantly altered the T-cell subsets and restored the CD4^+^/CD8^+^ ratio, improving cardiac function and quality of life [46]. Our study demonstrated a significant decline in CD4^+^/CD8^+^ in peripheral blood and spleen of HFHS diet-fed rats. However, BBR administration altered peripheral T-cell subsets and restored CD4^+^/CD8^+^ ratio to a near-normal status. This aligns with previous studies highlighting BBR as an effective immunomodulator [47,48]. Furthermore, CD4^+^ T cells play pivotal roles in mediating chronic inflammatory responses in obese mouse models [49]. Thereby, our results suggest that BBR may attenuate chronic inflammation state through regulating immune cell balance.

## 4. Materials and Methods

### 4.1. Animals

Male Sprague Dawley rats (8–10 weeks old, 300–400 g body weight) were procured from Beijing Vital River Laboratory Animal Technology Co., Ltd. (Beijing, China). The animals were maintained under standard laboratory conditions (2–3 rats per cage) with ad libitum access to food and water, in a temperature- and humidity-controlled environment. Following acclimatization, rats were randomly allocated into three experimental groups (*n* = 9 per group): NC group, HFHS group, and HFHS plus berberine (HFHS-BBR) group. The NC group was fed a standard chow diet, while both HFHS and HFHS-BBR groups received a specially formulated high-fat/high-sucrose diet (composition: 10% fat, 20% sucrose, 2.5% cholesterol, and 0.5% sodium cholate by weight) supplied by Beijing Keao Xieli Feed Co., Ltd. (Beijing, China) for a duration of 28 weeks. Starting from week 16 through week 28, the HFHS-BBR group was supplemented with 100 mg/kg body weight/day of berberine (BBR; Macklin, Shanghai, China), based on established protocols [19]. The experimental design timeline is presented in Figure 1A.

Body weight measurements were recorded at 4-week intervals throughout the study period. Upon completion of the experimental protocol, comprehensive assessments were performed, including evaluation of cardiac function, glucose tolerance, and blood pressure measurements. Following functional assessments, rats were anesthetized using 3–5% isoflurane (RWD, Shenzhen, China), and blood samples were obtained via abdominal aorta puncture. Immediately after sacrifice, heart, visceral adipose tissue and spleen were rapidly excised for subsequent biochemical analyses. Cardiac mass was precisely measured, and the heart index was determined by calculating the heart weight to body weight ratio (HW/BW).

All experimental procedures involving animals were performed in strict compliance with the ethical guidelines and regulatory standards established by the Chinese Council on Animal Care. The study protocol was approved by the Animal Care and Use Committee of the Chinese Academy of Medical Sciences (No. 00003917).

### 4.2. Echocardiographic Analysis

Cardiac functional assessment was performed at study termination utilizing transthoracic echocardiography with the Vevo2100 Imaging System (FUJIFILM VisualSonics, Toronto, ON, Canada). Rats were maintained under anesthesia with 2–3% isoflurane (RWD, Shenzhen, China) during the procedure. Comprehensive LV evaluation included measurement of LV mass, LVAW, EF, and FS using conventional M-mode echocardiography. Mitral valve inflow patterns were analyzed through pulse wave Doppler imaging to determine the E/A wave ratio. All parameter calculations were performed using VevoLAB software (Version 2.2.3) according to standard protocols.

### 4.3. Oral Glucose Tolerance Test (OGTT)

Following an overnight fasting period, rats received an oral glucose load of 2 g/kg body weight. Serial blood samples were obtained via tail vein puncture at predetermined time intervals (0, 15, 30, 60, 90, and 120 min) following glucose administration. Blood glucose concentrations were determined using a commercial glucose meter (Yuwell, Danyang, China). Glucose tolerance was quantitatively assessed by calculating the area under the curve (AUC) using GraphPad Prism 8.0 software for statistical analysis.

### 4.4. Blood Pressure Measurements

Systolic and diastolic blood pressure measurements were obtained non-invasively from conscious rats using the tail-cuff method with a CODA monitoring system (ADInstruments, Shanghai, China). Animals were individually restrained in temperature-regulated holders maintained at 37 °C, and hemodynamic recordings were performed under stable physiological conditions. For each measurement session, a minimum of ten readings were acquired and subsequently averaged to ensure data reliability.

### 4.5. Network Pharmacology

The collection of 407 targets associated with BBR were obtained from CTD (https://ctdbase.org, accessed on 4 March 2023), SwissTargetPrediction (www.SwissTargetPrediction.ch, accessed on 4 March 2023), and TCMSP (https://www.tcmsp-e.com/#/home, accessed on 4 March 2023) databases. The collection of 611 disease genes related to HFpEF was retrieved from the CTD and DiseGeNET (https://www.disgenet.org/, accessed on 4 March 2023) databases. The co-targeted genes were identified through taking intersection of BBR and HFpEF targeting genes. The protein–protein interaction (PPI) information of co-targeted genes was obtained from STRING (https://cn.string-db.org, accessed on 5 March 2023) database and visualized by Cytoscape software (Version 3.8.0). Functional and pathway enrichment analyses of 49 targets were performed using R programming language (Version 4.0.3) based on GO (http://geneontology.org/, accessed on 5 March 2023) and KEGG (https://www.kegg.jp/kegg/pathway.html, accessed on 5 March 2023) databases, presenting the results in bubble plots.

### 4.6. Biochemical Analyses

Creatine kinase (CK) activity and concentrations of brain natriuretic peptide (BNP), C-reactive protein (CRP), interleukin 1 beta (IL-1β), interleukin 6 (IL-6), and tumor necrosis factor alpha (TNF-α) were quantified using commercial ELISA kits (Elabscience, Wuhan, China) following the manufacturer’s protocols. Serum metabolic profiles, including lactate dehydrogenase (LDH) activity and levels of total cholesterol (TC), triglycerides (TG), low-density lipoprotein cholesterol (LDL-C), and high-density lipoprotein cholesterol (HDL-C), were determined using a Hitachi biochemical analyzer (Tokyo, Japan) in conjunction with commercial assay kits (BioSin Bio-Technology and Science Inc., Beijing, China). Myocardial tissue analyses included measurement of superoxide dismutase (SOD) activity and quantification of malondialdehyde (MDA), catalase (CAT), glutathione peroxidase (GSH-Px), and cyclic guanosine monophosphate (cGMP) using ELISA kits (Elabscience, Wuhan, China). Total nitric oxide (NO) production in myocardial tissue was assessed by determining nitrate and nitrite concentrations through a modified Griess reaction assay (Beyotime, Shanghai, China).

### 4.7. Hemogram Analysis

EDTA-anticoagulated blood samples were processed using the automated CELL-DYN^®^ CD3700 hematology analyzer (Abbott, Alameda, CA, USA) for complete blood count analysis. The system quantified white blood cell (WBC) populations, including neutrophil (NEU) and lymphocyte counts, along with platelet enumeration. The neutrophil-to-lymphocyte ratio (NLR) and systemic immune-inflammation index (SII) were subsequently calculated according to the formula provided in reference [20]:

NLR = [absolute neutrophils count (ANC)]/[absolute lymphocytes count (ALC)]

SII = [ANC × absolute platelets count (APC)]/ALC

### 4.8. Histopathological Analysis

Cardiac tissue samples were processed for histological analysis through fixation in 4% paraformaldehyde (PFA), paraffin embedding, and sectioning at 4 μm thickness. Tissue sections were subjected to H&E staining and Masson’s trichrome staining for morphological evaluation. Histopathological examination was performed using a Zeiss microscope (Shanghai, China), with digital image acquisition for quantitative analysis. Cardiomyocyte cross-sectional area (CSA) was determined from H&E-stained sections, while collagen volume fraction was assessed from Masson-stained sections, both quantified using ImageJ software (Version 1.53).

### 4.9. Immunofluorescence Analysis

LV tissue sections underwent sequential processing including deparaffinization, rehydration, and permeabilization. Following blocking with goat serum, sections were incubated with primary antibodies (1:200 dilution of rabbit anti-Titin polyclonal antibody, Bioss, Beijing, China) at 4 °C overnight. After PBS washing, sections were treated with appropriate secondary antibodies. Nuclear staining was performed using DAPI solution, followed by application of anti-fade mounting medium. Fluorescence imaging was conducted using a Zeiss microscope (Shanghai, China), and titin-positive areas were quantitatively analyzed using ImageJ software (Version 1.53).

### 4.10. RNA Isolation and qRT-PCR

Total RNA isolation from LV and visceral adipose tissues was performed using the PureLinkTM RNA Mini Kit (Thermo Fisher Scientific, Waltham, MA, USA), with subsequent quantification using NanoDrop spectrophotometry (Thermo Fisher Scientific, Waltham, MA, USA). Complementary DNA (cDNA) synthesis was carried out with the HiFiScript cDNA Synthesis Kit (CoWin, Taizhou, China). Quantitative reverse transcription polymerase chain reaction (qRT-PCR) analysis was executed on the CFX ConnectTM Real-Time PCR Detection System (Bio-Rad, Hercules, CA, USA) employing the UltraSYBR Mixture (CoWin, Taizhou, China). The specific primer sequences for target gene amplification are detailed in Table S1.

### 4.11. Western Blotting

Protein extraction from LV tissue was performed using RIPA lysis buffer (Thermo Fisher Scientific, Waltham, MA, USA). Protein samples (equal loading amounts) were separated on 8% SDS-PAGE gels and subsequently transferred onto PVDF membranes. Following 1 h blocking with 10% skimmed milk at room temperature, membranes were incubated with primary antibodies at 4 °C overnight, then with corresponding secondary antibodies for 2 h at room temperature. Protein band visualization was achieved using the BLT GelView6000 ProⅡ imaging system (Biolight Biotechnology, Guangzhou, China), with quantitative analysis performed using ImageJ software (Version 1.53). Comprehensive antibody information is provided in Table S2.

### 4.12. Flow Cytometry

After mechanical homogenization of spleen, splenic cells were filtered through a 300-mesh sieve. T lymphocytes were collected from blood and splenic cells using a rat lymphocyte separation kit (Solarbio, Beijing, China). Cells were washed, stained with antibodies specific to FITC-CD3, PerCP-eF710-CD4, and PE-CD8, and analyzed using a flow cytometer (Thermo Fisher Scientific, Waltham, MA, USA). Data analysis was performed using FlowJo software (Version 10.8.1). All antibodies were from Invitrogen (Thermo Fisher Scientific, Shanghai, China).

### 4.13. Statistical Analysis

All data were presented as mean ± SEM. Normality of data was assessed using the Shapiro–Wilk and Kolmogorov–Smirnov tests. The difference between two groups were examined with two-tailed paired and unpaired *t* tests. One-way ANOVA was used for multiple comparisons followed by Tukey’s post hoc test. All statistical analysis was conducted with GraphPad Prism 8.0. A *p* value < 0.05 was considered statistically significant.

## 5. Conclusions

In conclusion, this research has demonstrated that BBR markedly ameliorated cardiac diastolic dysfunction, decreased myocardial inflammation and oxidative stress, improved microvascular endothelial dysfunction, and reversed the pathological suppression of the NO/cGMP/PKG pathway in rats fed an HFHS diet. BBR also attenuated cardiac fibrosis and inhibited the TGF-β/Smads signaling pathway. Furthermore, BBR exhibited a broad spectrum of extracardiac benefits by ameliorating pro-inflammatory comorbidities, reducing systemic inflammation, and correcting immune dysregulation in rats with HFpEF. These findings collectively indicate that BBR exerts multi-beneficial effects on rats with HFpEF. Future research and clinical trials are essential to fully explain the molecular mechanisms mediating BBR’s therapeutic effects and to establish its clinical efficacy in HFpEF treatment strategies.

## Data Availability

The original contributions presented in the study are included in the article/Supplementary Materials.

## Supplementary Materials

The following supporting information can be downloaded at: https://www.mdpi.com/article/10.3390/ijms26104847/s1.
