# Supplementary material for: Exploring the Multi-Faceted Effects of Berberine in Ameliorating Diastolic Dysfunction in Rats with Heart Failure with Preserved Ejection Fraction
Source: Int J Mol Sci. 2025 May 19;26(10):4847. doi: 10.3390/ijms26104847 (PMC12112712; doi:10.3390/ijms26104847)
Supplement: Supplementary file 1 [file ijms-26-04847-s001.zip › ijms-3550559-supplementary.pdf]

Supplementary materials

Table S1. qRT-PCR primer sequences

**Gene**	**Forward（5'-3'）**	**Reverse（5'-3'）**
ANP	AGCCGAGACAGCAAACATCA	AGGTGGTCTAGCAGGTTCTTG
BNP	GAACAATCCACGATGCAGAAGC	GGGCCTTGGTCCTTTGAGAG
IL-1β	CCTATGTCTTGCCCGTGGAG	CACACACTAGCAGGTCGTCA
IL-6	TAGTCCTTCCTACCCCAACTTCC	TTGGTCCTTAGCCACTCCTTC
MCP-1	TTCTGGGCCTGTTGTTCACA	CAGCCGACTCATTGGGATCA
NF-κB	GATCGCCACCGGATTGAAGA	CTCGGGAAGGCACAGCAATA
VCAM-1	AGTTACACAGCAGTCAAATGG	CTTTCGGAGCAACGTTGAC
ICAM-1	AGGTATCCATCCATCCCACA	GCCACAGTTCTCAAAGCACA
NOX2	CTGCCAGTGTGTCGGAATCT	TGTGAATGGCCGTGTGAAGT
NOX4	CGTCCACCGTTACCAGACAA	TTGGCCTCTGCGTATTCGTT
Collagen I	GGAGAGAGCATGACCGATGG	AAGTTCCGGTGTGACTCGTG
Collagen III	AGGTCCAGGGATACGGGGTA	CAGGGAAACCCATGACACCA
α-SMA	CATCCGACCTTGCTAACGGA	AATAGCCACGCTCAGTCAGG
CTGF	GATGCACTTTTTGCCCTTCTTAATG	GAGTGGAGCGCCTGTTCTAA
TGF-β1	GTGGCTGAACCAAGGAGACG	AGGTGTTGAGCCCTTTCCAG
Smad2	ACCAGAAGGCATATAGGAAGG	CGCACTATCACTTAGGCAC
Smad3	AGGGCTTTGAGGCTGTCTACC	CCATTCAGGTGTAGCTCGATCC
TNF-α	GCCACCACGCTCTTCTG	GCAGCCTTGTCCCTTGA
GAPDH	TGATGGGTGTGAACCACGAG	GGCATGGACTGTGGTCATGA

Table S2. Detailed descriptions of the detected proteins

**Antibody**	**Species source**	**Cat. No.**	**Source**
GAPDH	Rabbit	AG8015	Beyotime, Shanghai, China
TNF-α	Rabbit	8184	CST, MA, USA
NF-κB	Rabbit	10745-1-AP	Proteintech, IL, USA
VCAM-1	Rabbit	ab134047	Abcam, Cambridge, UK
ICAM-1	Mouse	ab171123	Abcam, Cambridge, UK
NOX2	Rabbit	19013-1-AP	Proteintech, IL, USA
NOX4	Rabbit	14347-1-AP	Proteintech, IL, USA
p-eNOS	Rabbit	ab215717	Abcam, Cambridge, UK

eNOS	Mouse	ab76198	Abcam, Cambridge, UK
PKG	Rabbit	21646-1-AP	Proteintech, IL, USA
sGC	Rabbit	19011-1-AP	Proteintech, IL, USA
ARG-2	Rabbit	14825-1-AP	Proteintech, IL, USA
PDE5A	Rabbit	bs-2349R	Bioss, Beijing, China
Collagen I	Rabbit	AF7001	Affinity, OH, USA
Collagen III	Rabbit	AF0136	Affinity, OH, USA
TGF-β	Rabbit	21898-1-AP	Proteintech, IL, USA
p-Smad2	Rabbit	18338S	CST, MA, USA
Smad2	Rabbit	5339S	CST, MA, USA
p-Smad3	Rabbit	9520S	CST, MA, USA
Smad3	Rabbit	9523S	CST, MA, USA

Table S3. The 49 BBR-HFpEF intersecting genes

Gene name	Betweenness	Gene name	Betweenness
TNF	0.262830631	CTSL	0.005167859
PPARGC1A	0.111442671	COX4I1	0.004473368
MMP9	0.109415057	TYMS	0.003764808
NOS3	0.089396792	SIRT3	0.003409685
ACHE	0.081560284	VEGFC	0.00320489
CDKN1A	0.074828914	PPARD	0.002567228
BCL2L1	0.064129968	AURKB	0.002386464
FOXO3	0.06396862	NR3C2	0.002152989
PPARG	0.059684526	IL23A	7.45E-04
EDN1	0.056707263	FAS	7.40E-04
IL1B	0.045828371	XBP1	7.01E-04
MMP2	0.042455938	BNIP3	6.21E-04
DRD1	0.041666667	SPARC	1.27E-04
DPP4	0.041666667	BCHE	0
NAMPT	0.039471987	SI	0
PARP1	0.034482396	NR1I2	0
FABP4	0.018038915	CDKN1C	0
MAPK1	0.0135645	GRK5	0
ICAM1	0.01347174	ESRRA	0
H2AX	0.012272601	IGF2BP3	0
SPP1	0.010254233	TGFBR1	0
GSR	0.009202972	ALOX5AP	0
BBC3	0.007071539	CYBA	0
DHFR	0.005766047	LDLR	0
GATA3	0.005617905		

Table S4. Biological process through which BBR may exert anti-HFpEF effect obtained in GO enrichment

ID	Description	pvalue
GO:0007566	embryo implantation	1.86E-07
GO:0045923	positive regulation of fatty acid metabolic process	2.78E-06
GO:0045429	positive regulation of nitric oxide biosynthetic process	2.78E-06
GO:0150076	neuroinflammatory response	5.05E-06
GO:0030730	sequestering of triglyceride	1.09E-05
GO:0033189	response to vitamin A	1.31E-05
GO:0010888	negative regulation of lipid storage	2.45E-05
GO:0035066	positive regulation of histone acetylation	5.74E-05
GO:0090022	regulation of neutrophil chemotaxis	7.75E-05
GO:0060612	adipose tissue development	0.000130372
GO:0035094	response to nicotine	0.000188987
GO:0050873	brown fat cell differentiation	0.000231194
GO:0030574	collagen catabolic process	0.000246507
GO:0045058	T cell selection	0.000262462
GO:0002673	regulation of inflammatory response	0.000262462
GO:0051593	response to folic acid	0.000293578
GO:0010887	negative regulation of cholesterol storage	0.000293578
GO:0050872	white fat cell differentiation	0.000506341
GO:1901550	regulation of endothelial cell development	0.00087701

Table S5. Pathways through which BBR may exert anti-HFpEF effect obtained in KEGG enrichment

ID	Description	false discovery rate
hsa05418	Fluid shear stress and atherosclerosis	6.42E-09
hsa04668	TNF signaling pathway	2.72E-08
hsa04210	Apoptosis	1.89E-06
hsa05161	Hepatitis B	3.21E-05
hsa05323	Rheumatoid arthritis	4.07E-05
hsa04064	NF-kappa B signaling pathway	5.75E-05
hsa04659	Th17 cell differentiation	7.35E-05

hsa05321	Inflammatory bowel disease (IBD)	0.00021
hsa04932	Non-alcoholic fatty liver disease (NAFLD)	0.00032
hsa04657	IL-17 signaling pathway	0.00053
hsa04940	Type I diabetes mellitus	0.00078
hsa04115	p53 signaling pathway	0.0026
hsa05165	Human papillomavirus infection	0.0038
hsa04350	TGF-beta signaling pathway	0.0039
hsa04211	Longevity regulating pathway	0.0044
hsa04930	Type II diabetes mellitus	0.0155
hsa05221	Acute myeloid leukemia	0.0269
hsa04920	Adipocytokine signaling pathway	0.028
hsa04540	Gap junction	0.039
hsa04658	Th1 and Th2 cell differentiation	0.0395

Table S6. Hemogram analysis results

	**NC**	**HFHS**	**HFHS-BBR**
NEUs	1.20 ± 0.22	3.87 ± 0.76*	1.46 ± 0.18$^{\#}$
LYMs	2.16 ± 0.38	2.26 ± 0.08	2.71 ± 0.19
PLTs	829.80 ± 37.89	849.80 ± 49.52	905.40 ± 39.72
NLR	0.61 ± 0.12	1.66 ± 0.28*	0.54 ± 0.06$^{\#}$
SII	525.52 ± 121.88	1331.44 ± 141.12**	488.92 ± 58.04$^{\#\#}$

NEUs, neutrophils; LYMs, lymphocytes; PLTs, platelets; NLR, neutrophil to lymphocyte ratio; SII, systemic immune inflammation index. Data are expressed as the mean ± SEM. *P < 0.05, **P < 0.01 vs. NC group; $^{\#}$P < 0.05, $^{\#\#}$P < 0.01 vs. HFHS group.
